# Sustainable integration of digitalisation in nursing education—an international scoping review

**DOI:** 10.3389/frhs.2024.1344021

**Published:** 2024-04-11

**Authors:** Tim Tischendorf, André Heitmann-Möller, Sven-Nelson Ruppert, Maria Marchwacka, Sandra Schaffrin, Tom Schaal, Martina Hasseler

**Affiliations:** ^1^ Faculty of Health and Healthcare Sciences, University of Applied Sciences Zwickau, Zwickau, Germany; ^2^ Faculty of Healthcare, Ostfalia University of Applied Sciences, Wolfsburg, Germany

**Keywords:** digitalisation, professional care, education, digital competencies, sustainability—nachhaltigkeit

## Abstract

**Introduction:**

Trainees and teachers at nursing schools as well as nursing professionals are increasingly facing new challenges as a result of the digital transformation. Opportunities for the entire care system exist in the improvement of care quality and communication between those involved. However, this change also harbours risks, such as the use of immature digital applications in the care sector, data theft and industrial espionage. In order to be able to exploit the potential of digitalisation despite these risks, it is necessary to integrate relevant aspects such as digital skills into nursing training. The aim of this study is to investigate the extent to which the sustainable integration of digitalisation in nursing education is discussed.

**Methods:**

The methods of the systematic literature and database search were carried out in the form of a scoping review according to the PRISMA scheme. The PubMed and CINAHL databases were used for this purpose. The search period covered the years 2017–2023.

**Findings:**

After screening the titles and abstracts using inclusion and exclusion criteria, 13 studies were included in the synthesis of findings. The international literature focuses on content areas that highlight trends in digitalisation-related training in nursing. These focal points include concept development, considering the heterogeneity of demand constellations, as well as the reflexive reorientation of existing competences, whereby the technological competence of teachers is not disregarded. Other focal points relate to the initiation of digital skills in training and maintaining the employability of older nursing staff through professional development.

**Discussion:**

The literature research shows that there is a rudimentary discussion about digitalisation and curricular developments in nursing training in an international context, while the discourse in the German-language literature is less advanced. Among the sustainability desiderata derived from the literature is the involvement of nursing professionals in the development, testing and implementation of digital technologies. Only through active cooperation between nursing professionals and nursing sciences can the topic of digitalisation be integrated into the education and training of professional nursing in a targeted and future-oriented manner, whereby the focus should always be on the ability to deal with digital technologies and the associated change.

## Introduction

Health and healthcare systems around the world are undergoing change as a result of advancing digitalisation (1). Digital innovations in the healthcare system can make a decisive contribution to improving healthcare. In an international comparison, Germany is only making insufficient use of this digitalisation potential (1). Referring to a study by the Bertelsmann Foundation, it shows that many other European and Western countries are significantly more advanced in the application and use of digital applications in areas such as healthcare. The challenges in the context of digitalisation include not only compliance with data protection requirements, but also the availability and further development of digital skills and an overview of the variety of different technologies and approaches. With a view to the future challenges in professional care, the integration of digital possibilities into training is becoming increasingly important. Digitalisation offers the opportunity to make the care process more efficient by facilitating access to relevant information and optimising administrative processes (2). It also improves patient care by offering precise and individualised care, which also reduces the diverse workload of nursing staff (ibid.). Finally, there is a need to promote data-driven research and innovation in nursing science in conjunction with the potential of digitalisation, which in turn can lead to more advanced nursing practices and methods (3).

New technologies in nursing are discussed in numerous publications. However, the approaches to digitalisation in nursing vary widely, digital tools operate at different levels, and a current structured classification of development and digitalisation in nursing is hardly possible. A large part of publications deals with robotics and robotic systems (4, 5). The research literature reports on the use of service and logistics robotics in care contexts, social robotics, assistance robotics, mobilization robotics (4–7). Furthermore, the use of artificial intelligence in nursing is discussed (8). This can help improve the organisations of patient processes and treatment plans and/or provide all relevant information that physicians and nurses need to make correct decisions and/or help with repetitive or routine care tasks or medication management (3, 9). Other digital developments are being researched in the context of information and communication technologies, including projects such as telemedicine, telehealth, telenursing, computer-assisted documentation, or specific apps to support people with dementia (e.g., for cognitive stimulation) (2, 10, 11). Various publications also test digital monitoring and sensor technologies for behavioral analysis, fall and pressure ulcer prevention, or measurement of vital signs, etc. (12).

Opportunities for the entire healthcare system exist in the improvement of the quality of care as well as the communication between the stakeholders. However, this change also entails risks, such as the use of immature digital applications in the care sector as well as dealing with cyber attacks, data theft, and industrial espionage, which require an awareness of the sensitive data infrastructure. In order to exploit the potential of digitalisation despite these risks, it is necessary to integrate corresponding aspects into nursing training. The competencies and expertise of nurses in the use of digital technologies are therefore of central importance in order to integrate the roles, relationships, and responsibilities of all professional and personal groups involved in health care as well as ethical and professional dilemmas (13). As a result, nursing professionals need to acquire digital competencies in order to appropriately apply new digital tools in health care. Along with other basic skills such as reading, arithmetic, and writing, the European Union defines digital literacy as one of the eight key competencies for lifelong learning (14). According to Ferrari (15), digital literacy includes: knowledge, skills, and attitudes that enable people to use information and communication technologies and digital media to complete tasks, solve problems, communicate, manage information, create and share content, and thus create a knowledge base and use it appropriately, effectively, creatively, and at the same time critically, autonomously, and ethically. International studies (16, 17), among others, emphasize teachers’ technology acceptance in educational contexts. The focus should be on both the use of technology in teaching and the inculcation of critical reflective competence in nursing practice.

The aim of this article is to evaluate the discourse on the sustainable integration of digitalisation for nursing education in the field of nursing science. In doing so, possible connections, obstacles and perspectives resulting from the integration of digitalisation into nursing education, especially with regard to sustainability aspects, will be examined in detail. The primary research question aims to determine to what extent and in what form sustainable integration of digitalisation for nursing education becomes a topic in nursing science. In addition, the article aims to identify key areas that arise from these discussions and that may have implications for initial, continuing and further training in nursing as well as for nursing research.

## Methodology

The systematic literature and database search methodology followed the PRISMA scoping review process (18). The National Library of Medicine PubMed and Cumulative Index to Nursing and Allied Health Literature (CINAHL) databases were used for the search. Another hand search was conducted in the Educational Resources Information Center (ERIC) journal database, the Fachinformationssystem Bildung (FIS Bildung) [Specialised information system for education] of the Fachportal Pädagogik, the funding database of the German Research Foundation (GEPRIS), and the Federal Ministry of Health in Germany. A supplemental search was conducted using Google Scholar.

The database search was conducted in the period from March to April 2023. Various search strategies were implemented using appropriate search terms and Boolean operators. German terms and their English equivalents were used, with the terms occasionally shortened:

(Pflegewissenschaft OR nursing science) AND (Pflege OR nursing) AND (Digitalisierung OR digitalisation) AND (Nachhaltigkeit OR sustainability) AND (Vorteile OR advantages) AND (Nachteile OR disadvantages).

Studies from 2017 to 2023 were included and used flexibly according to the filtering capabilities of the databases to narrow down literature findings that were too large. The start of the search period in 2017 is based on the assumption that the research topics covered in the studies reflect the currently established use of digital technologies in care. This also justifies the lead time required for the published studies and meta-studies. The filters used were:

• set filters in PubMed: exclusion of preprints, studies in the period 2017–2023
• filters set in CINAHL: presence of abstracts, studies in the period 2017–2023

In the identification phase, 19,620 literature finds were organised in the PubMed and CINAHL databases and 931 literature finds were organised by hand searching additional sources using the Citavi 6 literature management and knowledge organisations program. A total of 20,551 sources were searched for duplicates using the program, and the duplicates were removed accordingly. This left us with 20,066 publications. A total of 152 publications were included in the preselection. The pre-selection from the 20,066 publications was made by reading the headings according to the predefined inclusion and exclusion criteria. In addition to the reference to the research question, studies that met the following criteria were included in the pre-selection (Table 1).

**Table 1:** Overview of the predefined inclusion and exclusion criteria.

Inclusion criteria	Exclusion criteria
Published language: • English or German	Published language: no English or German
Topics: • Reference to nursing • Reference to teachers at nursing colleges	Other topics: • no reference to nursing or nursing science • no relation to teachers at nursing colleges • focus on social media

**Figure 1:**
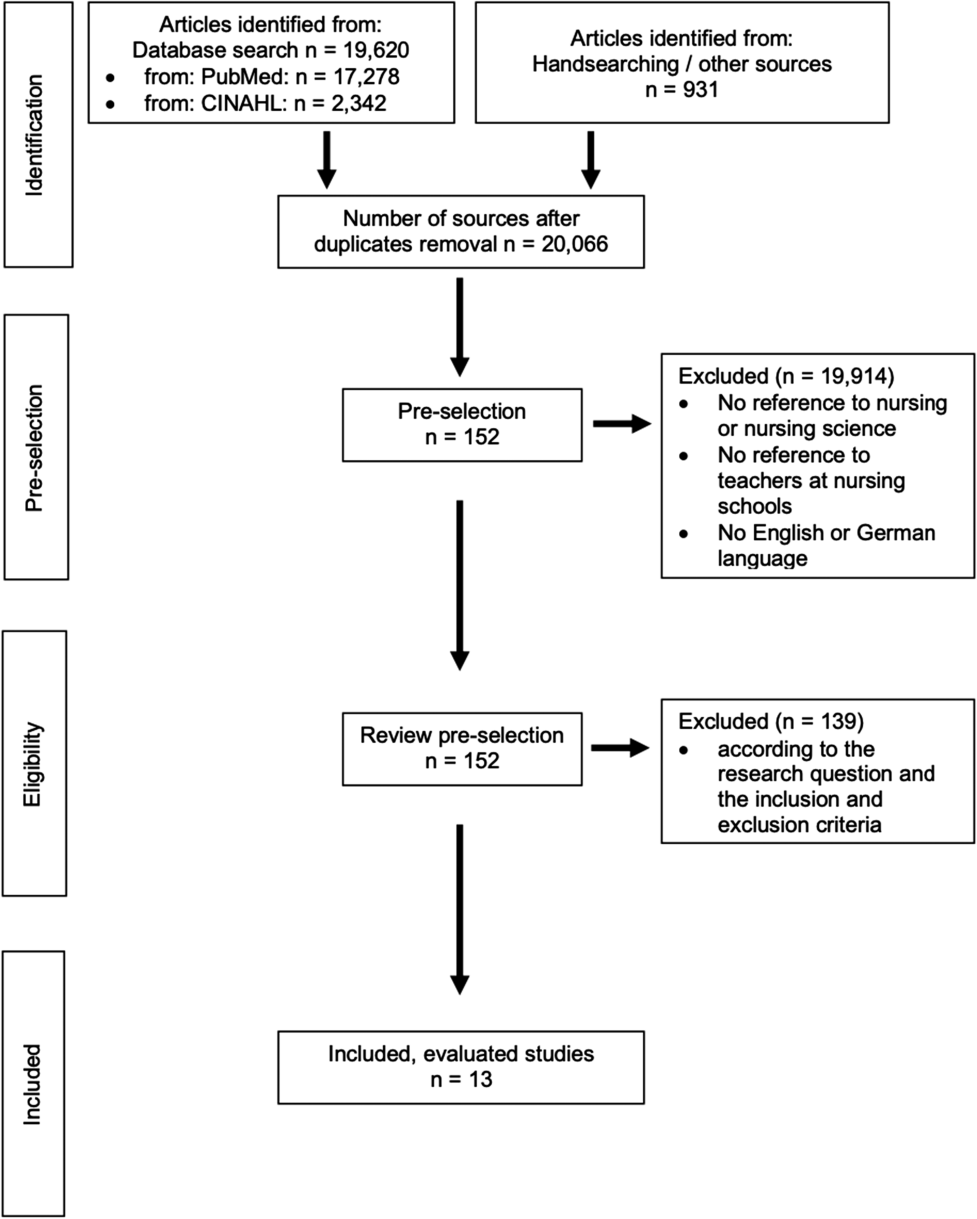
Flow chart of the literature search and selection based on (19).

After completing the pre-selection, the included texts were thoroughly assessed for their relevance to the research questions and adherence to the inclusion and exclusion criteria. Studies were excluded if they were written in neither German nor English and fell under the exclusion criteria in Table 1. In order to accomplish this, the full texts were downloaded from the respective databases or publishers' websites. Initially, abstracts were reviewed if they were available, and if no abstract was available, a cursory reading of the full text was conducted. The second step was the detailed reading of the full texts. Out of the 152 literature records included in the pre-selection, 13 sources were ultimately included in the analysis (Figure 1).

## Findings

A total of 13 studies were identified to answer the research question (Table 2). Of these, five were published in German and a further eight in English.

**Table 2:** Overview of included literature.

Study 1	Bleijenbergh, R., Mestdagh, E., Timmermans, O., van Rompaey, B., Kuipers, Y. J., 2023. Digital adaptability competency for healthcare professionals: a modified explorative e-Delphi study. In: Nurse education in practice 67, S. 103563. DOI: 10.1016/j.nepr.2023.103563.
Study 2	Brice, S., Almond, H., 2020. Health Professional Digital Capabilities Frameworks: A Scoping Review. In: Journal of multidisciplinary healthcare 13, S. 1375–1390. DOI: 10.2147/JMDH.S269412.
Study 3	Brown, J., Pope, N., Bosco, A. M., Mason, J., Morgan, A., 2020. Issues affecting nurses’ capability to use digital technology at work: An integrative review. In: Journal of clinical nursing 29 (15−16), S. 2801–2819. DOI: 10.1111/jocn.15321.
Study 4	Buhtz, C., Paulicke, D., Hofstetter, S., Jahn, P., 2020. Technikaffinität und Fortbildungsinteresse von Auszubildenden der Pflegefachberufe: eine Onlinebefragung [Affinity for technology and interest in continuing education among nursing trainees: an online survey]. In: HEILBERUFESCIENCE 11 (1/2), S. 3–12. DOI:10.1007/s16024-020-00337-5.
Study 5	Eiben, A., Mazzola, R., Hasseler, M., 2018. Digitalisierung in der wissenschaftlichen Weiterbildung im Bereich Gesundheit und Pflege. Herausforderungen und Chancen unter besonderer Berücksichtigung des Blended Learning Formates [Digitalisations in Continuing Academic Education in Health and Care. Challenges and opportunities with special consideration of the blended learning format]. In: Zeitschrift Hochschule und Weiterbildung (1), S. 31–37. DOI: 10.4119/zhwb-240.
Study 6	Evans, M., Kemper, J., Kucharski, A., Seyda, S., Hickmann, H., Pierenkemper, S., 2022. Gestaltungspfade und Gestaltungspraxis der Digitalisierung in der Altenpflege in NRW [Design paths and design practice of digitalisations in geriatric care in NRW]. Cologne (IW-Report. 2022,15). Online available at: https://ideas.repec.org/p/zbw/iwkrep/152022.html.
Study 7	Meissner, A., 2017. Technisierung der professionellen Pflege. Einfluss. Wirkung. Veränderung [Technicisations of professional nursing. Influence. Effect. Change.]. In Nomos Verlagsgesellschaft mbH & Co. KG eBooks, S. 153–172. doi: 10.5771/9783845279435-153
Study 8	Koch, D., 2021. Age Management in der ambulanten Pflege: Unterstützung älterer Pflegekräfte bei Digitalisierungsprozessen [Age Management in Outpatient Care: Supporting Older Caregivers in Digitalisationals Processes]. Gelsenkirchen (Institut Arbeit und Technik. Forschung aktuell. 2021,2). Online available at: http://hdl.handle.net/10419/231384; https://hdl.handle.net/10419/231384.
Study 9	Longhini, J., Rossettini, G., Palese, A., 2022a. Correction: Digital Health Competencies Among Health Care Professionals: Systematic Review. In: Journal of medical Internet research 24 (11), e43721. DOI: 10.2196/43721/ Longhini, J., Rossettini, G., Palese, A., 2022b. Digital Health Competencies Among Health Care Professionals: Systematic Review. In: Journal of medical Internet research 24 (8), e36414. DOI: 10.2196/36414.
Study 10	Mohr, J., Riedlinger, I., Reiber, K., 2020. Die Bedeutung der Digitalisierung in der Neuausrichtung der pflegerischen Ausbildung - Herausforderungen für die berufliche Pflege im Kontext der Fachkräftesicherung [The importance of digitalisation in the reorientation of nursing training - challenges for professional nursing in the context of securing skilled workers]. In: Evelyn Wittmann, Dietmar Frommberger und Ulrike Weyland (Hg.): Jahrbuch der berufs- und wirtschaftspädagogischen Forschung 2020. Opladen, Berlin, Toronto: Verlag Barbara Budrich, S. 165–182. Online available at: https://nbn-resolving.org/urn:nbn:de:0111-pedocs-206610.
Study 11	Gonçalves Nes, A. A., Steindal, S. A., Larsen, M. H., Heer, H. C., Lærum-Onsager, E., Gjevjon, R. E., 2021. Technological literacy in nursing education: A scoping review. In: Journal of professional nursing: Official journal of the American Association of Colleges of Nursing 37 (2), S. 320–334. DOI: 10.1016/j.profnurs.2021.01.008.
Study 12	Tacke, D., 2017. Chancen und Risiken computergestützter Pflegediagnostik [Opportunities and risks of computer-assisted nursing diagnostics]. In: Hagemann, T. (Hg.): Gestaltung des Sozial- und Gesundheitswesens im Zeitalter von Digitalisierung und technischer Assistenz. Baden-Baden: Nomos Verlagsges, S. 207-216. Online available at: doi: 10.5771/9783845279435-207
Study 13	Zelt, T., Weidner, F., Hülsken-Giesler, M., 2017. ePflege - Informations- und Kommunikationstechnologie für die Pflege [Information and communication technology for nursing]. Study on behalf of the Bundesministerium für Gesundheit. Cologne: Roland Berger GmbH, Deutsches Institut für angewandte Pflegeforschung e.V., Philosophisch-Theologische Hochschule Vallendar.

The Study 1 by Bleijenbergh et al. (20) aims to define elements of digital adaptability for healthcare professionals. Through an exploratory Delphi study, they identified a total of 29 topics covering personal attributes, interpersonal attributes and ethical aspects. Given the rapid progress in eHealth, healthcare professionals are expected to keep pace with digital developments. The study provides a concrete list of elements that reflect the digital adaptability skills of healthcare professionals and helps to transform the abstract concept of digital adaptability into a more pragmatic concept. These topics on digital adaptive competence according to Bleijenbergh et al. (20) are presented in Table 3.

**Table 3:** Items of personal characteristics, interpersonal characteristics and ethical aspects.

The healthcare professional…	
• - “…shows interest in eHealth”, • - “…uses eHealth for data retention”, • - “…uses eHealth as a supportive tool in care delivery”, • - “…uses eHealth as a tool to improve care”, • - “…is able to use the software/programs to access patient data”, • - “…feels confident in using eHealth to make care-related decisions”, • - “…is aware of the benefits provided by eHealth”, • - “…critically evaluates the reliability of data collected with eHealth applications”, • - “…supports the person in need of care to benefit from eHealth”, • - “…encourages the person in need of care to use eHealth”, • - “…adapts eHealth approaches according to the needs of the person in need of care”, • - “…behaves professionally in the use of eHealth”, • - “…is competent in the use of eHealth in relation to clinical reasoning”, • - “…feels that the use of eHealth in most situations improves the quality of life of the person in need of care”, • - “…has the skills to communicate with other health professionals via eHealth”, • - “…has the basic skills to use appropriate technologies such as the computer or a smartphone”,	• - “…knows where to reliably gather information using eHealth”, • - “…understands the impact of eHealth on improving the quality of health care”, • - “…communicates professionally with individuals seeking care through eHealth”, • - “…presents information gathered via eHealth in a manner that is understandable to individuals seeking care”, • - “…has skills via eHealth to communicate with individuals in need of care”, • - “…discusses the advantages and disadvantages of eHealth with individuals in need of care”, • - “…provides health-related counseling in the use of technology evidence-based care tools”, • - “…communicates in a professional manner with other health professionals via eHealth”, • - “…uses eHealth in personal routines”, • - “…proactively and regularly obtains consent from individuals in need of care to access personal information”, • - “…recognisess ethical dilemmas that may arise when integrating technology in health care and ethical principles.” • - “…discusses ethical dilemmas with the person in need of care that may arise due to ethical principles and the integration of technology in health care.” • - “…updates their knowledge of developments in health technologies through (continuous) learning”.

In Study 2 Brice and Almond (21) identified four topic areas, each containing three to four categories. The first topic area denotes change management with the categories of professionalism, education, professional standards, and non-technical skills. In the second topic area, “User Application”, three categories (User Development, Holistic Care, Participation) were identified. The third topic area includes the categories of Technological Skills, Technological Competence, and Managing Technology. The fourth topic area includes the categories of Innovative Practice, Innovative Behavior, and Applied Innovation. Overall, the authors conclude that Professional Digital Competency Frameworks require refocusing on currently known skills and competencies rather than rewriting them. They combine this approach with a focus on the identified topic areas and avoiding a one-sided perspective on skills/competencies isolated from one another (ibid.). These aspects need to be viewed holistically as interconnected aspects (ibid.).

In the third study, Brown et al. (22) were able to identify the following overarching themes from the included literature of their integrative review based on Whittemore and Knafl (33): 1. expertise and competence on the user side, 2. patient-centered access to data, 3. nurse concerns, and 4. investment in implementation. The top theme 1 links the factors that relate to nurses' informatics expertise in their clinical practice and can be associated with competency development (ibid.). The improvement of digital competencies in nursing is already addressed in nursing studies and through continuing professional education and training. However, the latter is less likely compared to the educational situation in the degree program for nurses working close to patients (34), cited by (22). The authors link access to evidence, on the one hand, and access to electronic health documentation, on the other hand, to the superordinate theme 2 (ibid.). Access to evidence-based information and data in electronic form facilitates decision making in the clinical context due to easy as well as efficient access to information in patient-centered care (ibid.). This is also reflected in access to electronic health documentation, as (almost) paperless documentation increasingly accompanies care (ibid.). The third overarching theme points to the fact that the impact of digital technologies on clinical outcomes is a key driver for their inclusion in the repertoire of actions (ibid.). However, there are concerns among nurses about the time-consuming use of digital technologies, which could limit the amount of time available for patient-centered care (ibid.). At the same time, stress and frustration are also associated with the use of digital technologies or their functionality, especially when the technology has not served its purpose (ibid.). In this case, nurses would revert to analog paper-based solutions (ibid.). Top topic 4 elaborates on the existing discourse in the literature regarding implementation. This refers to the removal of barriers or issues that may stand in the way of implementation (ibid.). Overall, Brown et al. (22) elaborated implications for practice, policy, and education in linking the themes. In terms of practice, 1. allocating time for nurses to explore and use digital platforms in patient-centered care, 2. offering help and support services for nurses in the event of technical glitches and problems, 3. offering patient-centered access to evidence-based information as well as support in identifying appropriate clinical information, 4. broad use of electronic records and documents as a means to improve quality and safety of care (ibid.). The implications for policy are formulated by Brown et al. (22) as follows: 1. involving nursing leaders, nursing informaticists, and clinical nurses in the development of digital platforms and systems, 2. developing policies regarding patient confidentiality and privacy as well as other sensitive information, and 3. a thorough implementation strategy with commitment and an appropriate time corridor for implementation of new technologies. With regard to education, five implications are elaborated (ibid.): 1. incorporation of curricular content to build digital literacy already established in education, 2. continuous professional development to build and maintain digital literacy in nurses, 3. separate time frame for orientation into the topic and (continuing) education in everyday professional life, 4. identification of support persons and their assignment to support the development of digital literacy in nurses, and 5. patient and family education with regard to the use and forms of digital technologies used in care.

The Study 4 by Buhtz et al. (23) conducted an online survey on the use of low-threshold technical solutions in the area of home care and the experiences in dealing with these at schools for geriatric and nursing care in the eastern German states. They found out that part of the trainees showed a strong willingness to participate in educational programs related to digital and assistive technologies. The respondents also saw a need for such offerings (ibid.). Accordingly, there is a high degree of openness to this topic, although their own knowledge was assessed as low (23, 35). However, concerning the integration of technical applications in the homes of individuals receiving nursing care, respondents had no difficulties in identifying these problems (23, 35). At the same time, most of the technologies for home use (medication dispenser, videophone, height-adjustable washbasins, daily calendar with reminder function) were unknown (23). Thus, from the authors' perspective, the imagination of the respondents is limited in the direction of beneficial use of technologies (ibid.). Overall, their perspective gives the impression that the ability to assess needs, the (competence-related) self-confidence in dealing with technologies and the integration of technologies should be questioned (ibid.). They justify this with the lack of knowledge about assistive systems and their inherent complexity (ibid.). Buhtz et al. (23) highlight that training programs and educational opportunities need to be integrated into training, as there are already care scenarios for technical aids. There is thus a need for the integration of teaching units on digital and assistive technologies into nursing education (23, 35).

In Study 5 Eiben et al. (24) interviewed a total of 32 nursing professionals participating in continuing education courses offered by various universities implemented in a blended learning format. In the evaluation results, the uncertainty of the target group becomes clear when it comes to the use of digital tools in the context of the events, such as the use of the learning platform, video tutorials, or even technical difficulties in setting up access to online-supported teaching. The results indicate that the participants tend to use learning strategies and examination formats they are familiar with from their learning backgrounds. In order to benefit from blended learning events, they need more support in individualisings their learning compared to “regular” students. However, under certain conditions, the integration of digital tools has advantages and can support the learning process. The evaluation identified several positive factors, including didactically prepared study material for the independent development of course content, integration of face-to-face events, offering technical support and support for independent or research-based learning, and assistance from online mentors.

Study 6 by Evans et al. (25) used a mixed methods approach to investigate current conditions and change trends regarding operational digitalisation processes in both outpatient and inpatient geriatric care. The results are summarised in three areas of tension: “Digital innovations in care,” “Need for digital competencies in geriatric care,” and “Shaping change processes together. In the case of “Digital innovations in care,” it is evident that not all technical possibilities have been fully utilised in the facilities thus far. Digital tools (employee files, roster management, etc.) are used comparatively frequently for organisationals and administrative tasks. In the context of nursing activities, digital systems (sensory systems, therapeutic robots, etc.) are used significantly less. The obstacles lie not only in financial aspects but also in the different framework conditions, which “lie outside the scope of action of the individual facilities” (ibid.). The field of tension “Need for digital competencies in geriatric care” is based on Becka et al. (36), who describe the competency areas and categorises them into 1. core competencies, 2. specialised competencies, and 3. reflexive and social-communicative competencies. In the context of the study, user competencies are most significant as core competencies, followed by reflexive and communicative competencies. According to the results, specialised competencies such as the operation of assistance systems play only a subordinate role. There is a desire for greater emphasis on teaching digital skills in vocational training, which is currently perceived as inadequate. At present, training in digital skills is mostly event-driven and focused on the specific use of a device or program. There is no systematic, target-group-specific identification of competence requirements that goes beyond user competence. In practice, little attention is paid to the last area of tension, “Shaping change processes together”. The results show that “although participation is understood as a core element for the successful organisations of change processes, it is not yet sufficiently implemented in practice and nursing staff only report feeling involved in decision-making processes to a limited extent. It is also clear that there is a broad spectrum of participation concepts in practice, which do not necessarily go hand in hand with the involvement of company interest groups. A stronger awareness of co-determination can possibly lead to a stronger identification of care workers with the company and increase employee loyalty to the company” (25).

The seventh study is a theoretical reflection article by Meissners (26), which first describes the development of technical and digital care in Germany from the 1950s to the present day. It then describes “new technologies of care” using the example of robotics, age appropriate assistance systems and information and communication technology. The consequences of the technical changes on the interactive relationship structure in care is not only accompanied by financial questions, but also by ethical questions. To address these concerns, a model for the ethical evaluation of socio-technical arrangements was developed in 2013 [MEESTAR, cf (37)], which “is intended as an analytical tool to guide reflection on the use of technology” (ibid., p. 17). The evaluation is carried out by combining seven ethical dimensions (care, self-determination, safety, justice, privacy, participation, self-image) with four levels of sensitivity. The model is to be applied in an interdisciplinary manner, involving all those involved in technology, from research to development and deployment. The model is differentiated into 15 guidelines, which are intended to support ethically oriented judgment, decision-making and action through questions (e.g., “Can care be delegated to technology?”) (ibid., p. 18). It is critically discussed to what extent technology, through its *per se* fixed parameters, opposes the negotiation process of care and how this creates limits in nursing studies. In the long term, the use of technology in nursing science can only succeed if it is more strongly anchored in education, receives greater relevance in research, a “systematic integration in theory building” takes place, and a “society-wide debate about the notion of nursing dependency and the use of technology” is initiated (ibid., p. 25).

Study 8 by Koch (27) uses a literature analysis and qualitative survey of four exemplary interviews to examine the question of how age management should be designed in outpatient care in order to support older caregivers as digitalisation continues to increase. The existing discussion on the effects of digitalisation on care activities and organisations is expanded with the perspective of age management, which has received relatively little attention. Through literature analysis and qualitative survey, the possible uses and applications of digital technology were investigated. Electronic care documentation, electronic tour planning, digital duty scheduling and service recording proved to be suitable for the field of outpatient care. The prerequisite for the use of the technologies is, of course, the corresponding competences of the care workers. Some measures of age management proved to be helpful to support older care workers in outpatient care in countering the effects of digitalisations in the area of care planning and care process organisation. Especially actions related to the dimensions of “awareness/sensitisations”, “(age) diversity” and “changed/changing attitudes” can have a positive impact on the inclusion of (older) employees in the introduction and use of digital technologies. Lifelong and shared learning, regardless of age, can contribute to increasing age diversity, employee motivation and knowledge transfer. According to the author, supervisors and managers play a central role, as the measures of age management must come from them.

Longhini et al. (28) identified four areas of research from the literature included in a systematic review analysing the health literacy of health professionals in Study 9: 1. studies on self-assessed competencies, i.e., studies on digital literacy, eHealth competency, patient-centered competencies and the process of care-oriented competencies. 2. studies on psychological and emotional aspects towards the use of digital technologies, i.e., attitudes and beliefs, trust, awareness. 3. studies on the use of digital technologies, i.e., digital literacy, eHealth competence, patient-centered competencies and the process of care-oriented competences. 3. use of digital technologies, i.e., general use of digital technologies, use of digital technologies for specific functions, and 4. knowledge about digital technologies. In summary, the authors noted an increase in the number of studies over the last five years, with most studies having a cross-sectional design or examining frequencies. Only two studies had an experimental design. About half of the studies focused on the hospital setting, making the community level less prominent. All professional groups were covered in the studies. At the same time, the authors identified a need for a stronger conceptualisations of the studies. A lack of validated instruments to measure digital literacy was also noted. It is assumed that the already existing instruments in the respective studies can be used to a limited extent and the rapid evolution of digital technologies requires continuous updates of competencies and, consequently, the corresponding measuring instruments. Furthermore, self-assessments were made in all studies, thus lacking objective measurements. The authors, therefore, call for the increased use of objective measurements by third parties, which, however, have yet to be developed in the research field.

In Study 10, Mohr et al. (29) describe a desideratum with regard to basic and applied research on the topic of digitalisation in nursing. However, it is not only academia that pays little attention to the topic. Digital technologies also play a rather minor role in nursing practice and vocational training. In the Nursing Professions Act, the teaching of digital skills has so far only been mentioned in connection with university education, but this is clearly not enough for everyday nursing practice. Beyond the Nursing Professions Act, some concrete forms of application can be found in various framework curricula. However, which concrete competences are required for application-related use in practice have so far been mentioned neither in the law nor in the curricular training. “In the course of a further profiling of the professional profile, it would be necessary to grasp digitalisations as an integral component: Not merely as a further (possibly annoying) content add-on in an already extensive content catalogue, but as a cross-sectional competence to the nursing core and contextual competence under the condition that the professional nurses define and control the use of digitalised technology” (ibid., p. 178). The implementation should accordingly take place with the involvement of the stakeholders; here, clear parallels emerge in the articles by Mohr et al. (29) and Evans et al. (25), which also call for a more participatory approach to the recording and definition of digital competences from practice. Accordingly, the development would take place less from the subject-specific and technically possible point of view but could rather be developed and implemented individually from the professional logic. The prerequisite here is to define and systematically clarify the concepts of digitalisations/digital technologies in order to develop a common basic understanding from practice for practice.

Gonçalves Nes et al. (30) conducted a scoping review on technology competence in nursing education in Study 11. They grouped the 28 included studies according to their thematic focus. Eight publications focused on the acquisition of technical knowledge and skills. The second thematic focus of the remaining 21 publications was the measurement of technical knowledge and skills (ibid.). Here, the authors distinguished between sub-topics, with some of the publications addressing several sub-topics (ibid.): 10 publications addressed digital/computer literacy/competence, 9 publications examined nursing informatics literacy, 2 examined technology acceptance, and 4 examined students’ technology-related interests and preferences. A single publication related to the thematic focus of maintaining technical knowledge and skills (ibid.). Thus, the scoping review aimed to map and explore the topic of technical literacy in nursing education. The measurement instruments used in the included studies showed a high degree of heterogeneity, which underlines the need for a universally applicable instrument to achieve comparable results (ibid.). Gonçalves Nes et al. (30) derive several implications from their scoping review: First, nurses need to take a more active role in technology development and implementation, including claiming a leadership role. Relevant to building strategies for acquiring technical literacy is the availability of information about nursing students’ technology acceptance and interests, as well as their preferences. It is essential to implement strategies that increase technical literacy among teaching staff, enabling them to successfully impart this literacy to students. The findings suggest the need for more knowledge to maintain technical literacy. This in turn requires an increase in IT competences and skills among teachers, based on which appropriate strategies as well as methods can be defined.

As part of Study 12, Tacke (31) deals with the risks and opportunities associated with computer-aided nursing diagnostics in her theoretical reflection contribution. The advantages of digital technologies, such as standardisation of processes through nursing documentation systems and ensuring quality, are obvious. However, if these systems are to be used across the board for nursing diagnostics, extensive training and further education of the nursing staff is required in order to perform the translation from pure documentation to validated diagnostics. Various other studies (38–40) show that nurses have so far been insufficiently prepared for this process leading to diagnostics and have problems “observing specifically, interpreting what is perceived and taking the patient's perspective” [(31), p. 212]. Further training in the use of digital technologies should therefore not only refer to the purely technical teaching, but also sensitises for the practical use and stimulate and promote the critical reflection ability of the nursing staff (ibid.).

The Study 13 “ePflege” was commissioned by the Federal Ministry of Health (BMG) and was conducted as a mixed methods study to analyse the current status quo and the resulting identification of needs and the development of recommendations for action. The results indicate that while digitalisations is of great importance, the focus on networked care and the inclusion of users has not been in the foreground so far. Associated expectations of care using digital solutions are above all quality improvement, increased efficiency, the reduction of bureaucracy and the networking of actors. The lack of technical skills on the part of the users and the exchange of information among each other are usually obstacles to the use of digital solutions. If existing digital solutions exist, they are not considered very user-friendly, and the securing of long-term financing for these solutions is also often unclear (ibid., p. 7). In order to promote the reputation, the effects of digital techniques on the care process and their implementation and benefit assessment from an application perspective would be of interest. However, there is a clear need for research in this area as well. The respondents, differentiated according to various stakeholder groups, highlighted different focal points of need. Beneficiaries seek greater involvement in technology development and better information exchange. Careers expect greater involvement in implementation and better networking among themselves, while developers of digital technology solutions would like to see an improvement in the technical infrastructure and greater involvement of users in the development and implementation process (ibid., p. 8). “To promote the anchoring of care ICT in the health care system, the study recommends the establishment of a “Network ICT in Care”, the creation of incentives for the widespread use of electronic care documentation and the strengthening of transparency about the benefits of ICT solutions for anchoring in the initial care market” (ibid., p. 8).

In order to provide a more differentiated insight into the findings of the studies in relation to the research question, they have been summarised in a table (Table 4).

**Table 4:** Overview of the findings in relation to the research question.

Study (year of publication)	Topic	Method	Findings in relation to the research question
Study 1—Bleijenbergh et al. (20)	Digital adaptability competency for healthcare professionals	Explorative e-Delphi study	The 29 identified themes of digital adaptive competence provide a comprehensive insight into the diverse requirements and challenges associated with the use of digital technologies in nursing education. Consideration of these topics is crucial to ensure that the integration of digitalisation is sustainable and that both individual needs and ethical standards in nursing education are taken into account.
Study 2—Brice and Almond (21)	Health Professional Digital Capabilities Frameworks	Scoping Review	Based on the identified subject areas and categories and the conclusion of Brice and Almond (21), it can be stated that it is not sufficient to adapt or redefine individual skills or competences for a sustainable integration of digitalisation into nursing education. Rather, a holistic approach should be pursued that links the various subject areas such as change management, user applications, technological skills and innovative practice. Professional digital competence frameworks must therefore not only map the current skills and competences, but also focus on the identified subject areas in order to ensure a holistic and sustainable integration of digitalisation into nursing education.
Study 3—Brown et al. (22)	Issues affecting nurses’ capability to use digital technology at work	Integrative review	The authors emphasise the importance of a holistic view that takes into account personal, interpersonal and ethical issues in addition to technical aspects. Their conclusions suggest that the discussion on the sustainable integration of digitalisation should include not only technical, but also practical, political and educational aspects. Implications for practice include the provision of time and support for nurses and patient-centred access to evidence-based information. At a policy level, the involvement of various stakeholders in the development of digital platforms and privacy policies is emphasised. In education, the integration of digital competences into curricula and continuing professional development is emphasised.
Study 4- Buhtz et al. (23)	Affinity for technology and interest in continuing education among nursing trainees	Onlinesurvey	The authors emphasise the need to integrate training courses on digital and assistive technologies into nursing education in order to meet the needs of practitioners and strengthen confidence in the use of technology in the long term.
Study 5—Eiben etal. (24)	Digitalisation in Continuing Academic Education in Health and Care—Special consideration of the blended learning format	Interviews	Overall, the results show that the discussion about the sustainable integration of digitalisation into nursing training encompasses both challenges and opportunities and requires careful adaptation of teaching and learning methods to effectively support the learning process.
Study 6 -Evans et al. (25)	Design paths and design practice of digitalisation in geriatric care in NRW	Mixed-Methods-Design	The study highlights the complex areas of tension associated with the sustainable integration of digitalisation into nursing education. Firstly, it is found that digital innovations in care are not yet widely used, especially in nursing activities, partly due to financial barriers and external conditions. Secondly, a high need for digital competences in nursing is identified, with user competences in particular being considered crucial. However, the teaching of these competences is considered insufficient and is mostly event-driven. Thirdly, the need to jointly shape change processes in practice and promote the participation of care staff is emphasised in order to strengthen their identification with the company.
Study 7—Meissner (26)	Technicisation of professional nursing	Theoretical reflection	For the sustainable integration of digitalisation in nursing education, Meissner (26) emphasises the importance of ethical considerations in the integration of digital technologies in nursing, in addition to the comprehensive integration of technologies in nursing education.
Study 8—Koch (27)	Age Management in Outpatient Care: Supporting Older Caregivers in Digitalisation Processes	Literature analysis and qualitative interviews	In connection with the sustainable integration of digitalisation in nursing training, it can be concluded from the study that the recognition of age diversity and the teaching of age management measures can represent considerable added value in training.
Study 9—Longhini et al. (28)	Digital Health Competencies Among Health Care Professionals	Systematic review	Longhini et al. (28) note that the discussion about the integration of digital technologies into nursing education is increasing. There is a need for better conceptualised studies and validated measurement tools for digital competences.
Study 10—Mohr et al. (29)	The importance of digitalisation in the reorientation of nursing training—challenges for professional nursing in the context of securing skilled workers	Delphi survey	The study by Mohr et al. (29) emphasises a deficit in research and practice with regard to the integration of digital technologies into nursing training. It is noted that digital competences are insufficiently taken into account in the Nursing Professions Act and are not sufficiently defined in the curricula. It is recommended that digitalisation be considered an integral element of the nursing profession and that the development of digital competences be driven forward in a participatory manner with the involvement of those affected.
Study 11—Gonçalves Nes et al. (30)	Technological literacy in nursing education	Scoping Review	The study by Gonçalves Nes et al. (30) shows that nurses should take a more active role in technology development. Teachers need to improve their IT competences in order to teach technical skills effectively.
Study 12—Tacke (31)	Opportunities and risks of computer-assisted nursing diagnostics	Theoretical reflection	For the sustainable integration of digitalisation into nursing education, Tacke (31) emphasises the importance of the training and further education of nursing staff for the use of computer-aided nursing diagnostics. It becomes recommended to focus further training not only on technical use, but also on practical application and critical reflection.
Study 13—Zelt et al. (32)	Information and communication technology for nursing	Mixed-Methods-Design	For the sustainable integration of digitalisation into nursing education, it is crucial to focus on networked care and the involvement of users. Nursing training must therefore concentrate more on teaching digital competences and also take into account the needs and perspectives of different stakeholder groups.

## Discussion

From the overall view of the literature included, it becomes clear that the sustainable integration of digitalisation in nursing education is a topic of discussion in the nursing science discourse. A differentiation can be made between different key areas. One key area is concept development. This includes the orientation towards pragmatic concepts (20) as well as the consideration of the heterogeneity of the demand constellations [with reference to (32) and the previously existing competences in the sense of a reflexive refocusing (21)]. Instead of looking at existing competences and skills in isolation and trying to circumscribe them, these must be viewed holistically as interconnected aspects (21).

Another key area relates to the initiation of digital competences in training. Brown et al. (22), Buhtz et al. (23) and Meissnerss (26) emphasises the importance of laying the foundations for digital (technical) competences in basic nursing training. However, Brown et al. argue in the context of fully academicised nursing training, which has not yet been established in Germany, for example. In order to classify the results of Buhtz et al., the fallacy can be raised here that the structured acquisition of digital competences could become obsolete in the future due to the generation of so-called digital natives. Just because a person has grown up surrounded by computers, cell phones, and other digital devices does not automatically make them digitally competent. Their informally acquired skills to use technology safely and effectively are likely to be incomplete (41). This leads to a new digital divergence between digital lifestyle skills and digital workplace skills. It means that young people are not automatically excluded from digital skills education (41). Implications for the long-term use of technology in nursing education include a stronger integration of technology into nursing education, greater relevance in research, systematic integration into theory development and a societal debate on the concept of the need for care and the use of technology (26).

Other key areas in the discourse are vocational training (24, 31) and participatory technology development (25, 29). Further training to acquire digital competence currently takes place mostly on an *ad hoc* basis and with a view to the specific application of a device or program. There is no systematic and target group-specific identification of competence needs beyond user competence (25). For the further profiling of the professional profile, digital competences should not be a content-related add-on in a complex education, training and continuing education catalogue, but should be regarded as an integral component of a core or cross-sectional competence of professional nurses (29). In line with the emphasis on participatory technology development, Gonçalves Nes et al. (30) call for nurses to take an active role in technology development and implementation, and recommend that leaders actively support this process. Participatory technology development can thus make a decisive contribution to the sustainable implementation of digitalisation in nursing.

Other publications refer to the topics of maintaining the working ability of older nursing professionals (27), the adaptation of competence development to the evolution of digital technologies (28) as well as the technology literacy of teaching staff (30). Action-oriented age management measures can therefore positively impact the inclusion of (older) employees in the introduction and use of digital technologies. Supervisors and managers play a central role in such age management orientation (27). Building strategies for acquiring technical literacy goes hand in hand with information about students' technology acceptance, interests and preferences (30). The development of corresponding strategies could be done by teachers, although this requires a certain level of technical expertise. Early incorporation of this age management orientation during nursing education can therefore result in a sustainable strategy for the integration of digitalisation into nursing care. In doing so, trainees should become familiar with the concept of age management in order to subsequently achieve effective empowerment of older staff in practice by promoting the acceptance of technology.

Based on the literature, it is evident that digitalisations is a topic under discussion. Sustainable implementation appears more likely in an international context than in Germany. The following sustainability desiderata can be derived from the aforementioned key points in a broad sense: a high need for (basic) research activities, the conceptual design of competence attributions, the integration of nursing science expertise in the drafting of curricula and, above all, the integration of nursing professionals in the development, testing and implementation of digital technologies. In the context of the high demand for research activities, Longhini et al. (28) call for the development and subsequent inclusion of objective measurement tools related to the use of digital technologies and associated digital competences. A first starting point is the DigComp Framework of the European Union (42). The DigComp framework identifies the key components of digital literacy in the following 5 areas: information and data literacy, communication and collaboration, digital content creation, safety, problem solving (42).

The discussion about the sustainable integration of digitalisation into nursing education raises a number of open research questions. These include the development of teaching concepts, the measurement and teaching of digital competences, participatory technology development, age management in the context of technology acceptance and long-term strategies for the use of technology. These questions reflect the complexity and the various dimensions that need to be considered when integrating digital technologies into nursing education and offer important starting points for future research activities and the development of educational concepts in this area.

In the context of this scoping review, it is important to note that the work does not claim to cover the topic in its entirety. This is explained by the underlying research question on one hand, and by the search operators and search filters on the other. A further limitation arises from the literature itself, as it mainly provides findings at a meta-level. In this sense, this review already relies on third party interpretations. Nevertheless, the work offers added value by addressing the discourse on the sustainable integration of digitalisation in nursing education in the context of nursing science.

## Data Availability

The original contributions presented in the study are included in the article/Supplementary Material, further inquiries can be directed to the corresponding author.
